# Enhancing elevated temperature strength of copper containing aluminium alloys by forming L1_2_ Al_3_Zr precipitates and nucleating θ″ precipitates on them

**DOI:** 10.1038/s41598-017-11540-2

**Published:** 2017-09-11

**Authors:** Surendra Kumar Makineni, Sandeep Sugathan, Subhashish Meher, Rajarshi Banerjee, Saswata Bhattacharya, Subodh Kumar, Kamanio Chattopadhyay

**Affiliations:** 1 Indian Institute of Science, Department of Materials Engineering, Bangalore, 560012 India; 2 Indian Institute of Technology, Department of Material Science and Metallurgical Engineering, Hyderabad, 502285 India; 3 University of North Texas, Center for Advanced Research and Technology and Department of Materials Science and Engineering, Denton, TX-76203 USA; 4 Max-Planck-Institut für Eisenforschung, Department of Microstructure Physics and Alloy Design, Düsseldorf, 40237 Germany

## Abstract

Strengthening by precipitation of second phase is the guiding principle for the development of a host of high strength structural alloys, in particular, aluminium alloys for transportation sector. Higher efficiency and lower emission demands use of alloys at higher operating temperatures (200 °C–250 °C) and stresses, especially in applications for engine parts. Unfortunately, most of the precipitation hardened aluminium alloys that are currently available can withstand maximum temperatures ranging from 150–200 °C. This limit is set by the onset of the rapid coarsening of the precipitates and consequent loss of mechanical properties. In this communication, we present a new approach in designing an Al-based alloy through solid state precipitation route that provides a synergistic coupling of two different types of precipitates that has enabled us to develop coarsening resistant high-temperature alloys that are stable in the temperature range of 250–300 °C with strength in excess of 260 MPa at 250 °C.

## Introduction

The amount of strengthening of the Al matrix through dispersion of hard second phase intermetallics depends on the nature of intermetallic/matrix interfaces (coherent or incoherent), hardness of the intermetallic phases, their stability at high temperatures and size and volume fractions^1–7^. The types of intermetallic to be dispersed and their distribution depends on the alloying additions, heat treatment employed and mechanical working^8–11^. The present work is influenced by the recent reports on the development of core-shell precipitates in Al-Li-Sc^12^ and Al-Zr-Sc^13, 14^ ternary alloys that have resulted in an increase in stability at high temperatures. The precipitates in binary Al-Sc, Al-Li and Al-Zr have L1_2_ ordered crystal structure with the stoichiometry Al_3_Sc (stable), Al_3_Li and Al_3_Zr (both metastable) respectively that share a coherent interface with the fcc α-Al matrix. In ternary Al-Li-Sc alloy, a two-step ageing has resulted in the formation of a shell of Al_3_Li on Sc-rich precipitates yielding a narrower particle size distribution that delays the onset of particle coarsening. Similarly, in Al-Sc-Zr alloy, the precipitates have core mostly rich in Sc with an external shell, which is Zr-rich that provides better resistance to coarsening. However, the high cost of Sc and its limited availability restricts their use in commercial Al alloys. In addition, these precipitates provide limited room temperature strength due to their inability to form in high volume fractions unlike copper rich θ′/θ″ (in Al-Cu based alloys) or Zn/Mg-rich η′ (in Al-Zn-Mg based alloys) precipitates^15^. The alloys containing copper, in particular, the 2219 and 2618 alloys belonging to the 2XXX series of aluminium alloys, exhibit thermal stability at relatively high temperatures. However, precipitates in these alloys (θ′/θ″) coarsen rapidly at temperatures >150 °C resulting in a steep loss of strength^16^. The addition of Ag and Mg to 2219 alloy has been shown to form of Ω precipitates on {111} planes instead of θ′ on {100} planes. The Ω phase with an orthorhombic crystal structure is relatively more stable up to 200 °C due to the presence of layers of Mg and Ag atoms at the surface of the Ω plate-shaped precipitates. These restrict the formation of ledges thereby decreasing kinetics of coarsening at temperatures up to 200 °C^17, 18^.

We present a new approach in designing an Al-based alloy through conventional solid state precipitation route that provides a synergistic coupling of two different types of precipitates that can overcome the 200 °C barrier. The novelty in the current microstructure design is to heterogeneously nucleate low temperature strengthening precipitates of copper (θ′/θ″) on high-temperature Al_3_Zr precipitates having L1_2_ ordered structure that leads to resistance to coarsening of the microstructure. We have utilized a significant difference in temperatures required for nucleation/formation of these precipitates from the solute supersaturated α-Al to design the microstructure through a new three step heat treatment. A small amount of Nb was added to avoid discontinuous precipitation^19^.

## Results

### 3-Step heat treatment and subsequent microstructural evolution

The as-cast alloy (Al-2Cu-0.1Nb-0.15Zr in at.% everywhere) was subjected to a unique three step heat treatment. It comprised of direct ageing of the as-cast alloy at 400 °C to form the ordered Al_3_Zr precipitates. This was followed by an optimized higher temperature solution treatment at 535 °C for 30 minutes to dissolve copper solute in the aluminium matrix without affecting the Al_3_Zr precipitates. The alloy was then aged at 190 °C to precipitate mainly θ″ along with few θ′ precipitates heterogeneously on the preexisting Al_3_Zr precipitates.

**Figure 1:**
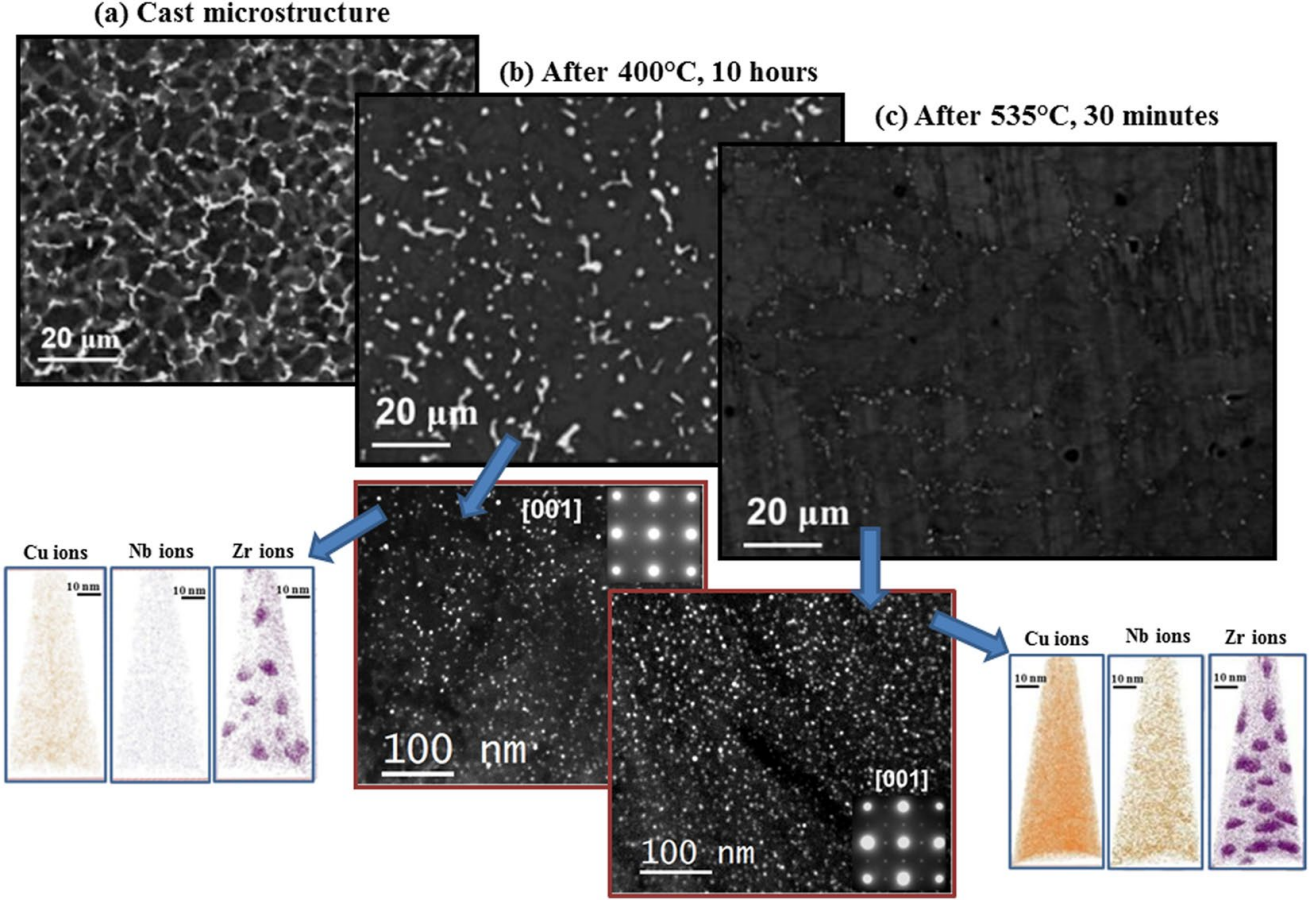
(**a**) Back scattered electron (BSE) micrograph of cast Al-2Cu-0.1Nb-0.15Zr alloy. BSE micrographs and TEM darkfield micrographs near to [001] zone axis using 100 superlattice spot reflecting L1_2_ spherical precipitates and individual ion maps from atom probe tomography (**b**) after aging at 400 °C for 10 hours and (**c**) subsequently after solutionising at 535 °C for 30 minutes.

Figure 1(a) shows the cast structure that exhibits a cellular microstructure with the evidence of solute segregation at the boundaries. Elemental mapping (shown in Figure S1) using wavelength dispersive spectroscopy (WDS) reveals enrichment of Cu along the cell boundaries with Nb and Zr partitioning inside the cells. The hardness of the as-cast alloy was found to be 740 ± 12 MPa. After ageing at 400 °C for 10 hours (peak ageing, Figure S2a), the microstructure has still copper-rich region in the cell boundaries, as shown in Fig. 1(b). The selected area diffraction (SAD) pattern from the cell interior along [001] zone axis (shown as inset) contains superlattice spots corresponding to the L1_2_ ordering along with the main fcc α-Al reflections. The darkfield micrograph taken from the 100 superlattice spot reveals a homogenous distribution of the ordered spherical precipitates inside the cells. The precipitates have an average size of ~5 nm. These ordered spherical precipitates were found throughout the microstructure without any discontinuous precipitation. Chuan and Tu^19^ have shown that the addition of Nb to Al-Zr based alloy minimizes the lattice misfit and interfacial energy that reduces the tendency to discontinuous precipitation of L1_2_ Al_3_Zr. From the atom probe measurements, the spherical precipitates are found to be Zr-rich with small enrichment in Nb and Cu with respect to the α-Al matrix (Figure S2b). The total solute content in the ordered precipitates determined from atom probe tomography (Al-2.03Cu-0.5Nb-22Zr) is 24.53 at.% that is close to the stoichiometry Al_3_Zr with minor amounts of Cu and Nb replacing Zr.

Subsequent to the peak ageing at 400 °C, the alloy was subjected to solutionising at 535 °C (below the Al-Cu eutectic temperature to avoid incipient melting) for 30 minutes followed by quenching in water to obtain supersaturated solid solution (SSSS) at room temperature. The microstructure of the alloy after solutionising shows (Fig. 1(c)), that copper is homogenously distributed in the Al matrix (see WDS mapping, Figure S3). However, the darkfield micrograph taken from 100 superlattice reflection reveals spherical L1_2_ ordered precipitates (Zr rich, Figure S4a) with sizes in the range of ~5 nm, similar to that obtained before solutionising the alloy.

**Figure 2:**
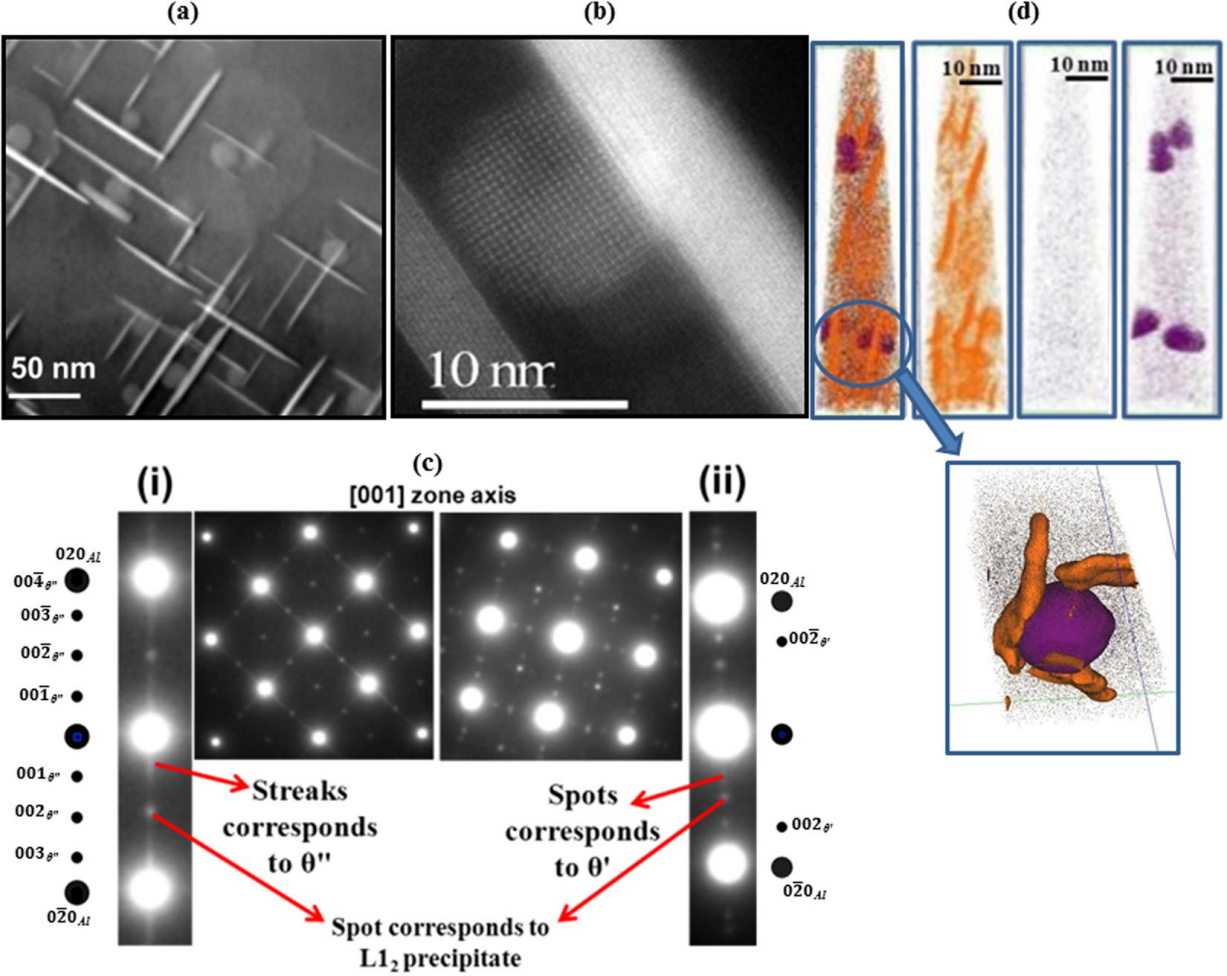
Microstructural details of Al-2Cu-0.1Nb-0.15Zr alloy after final step aging at 190 °C for 5 hours. (**a**) STEM HAADF micrograph and (**b**) high resolution micrograph taken near to [001] zone axis. (**c**) Diffraction patterns along [001] zone axis and (**d**) individual ion maps from atom probe tomography.

After solutionising, the alloy was artificially aged at 190 °C and hardness vs time plot is shown in Figure S4b. It was also compared with that obtained from the binary as-cast Al-2Cu alloy that was directly solutionised at 535 °C for 30 minutes and artificially aged at 190 °C. The hardness increment for the Zr-containing alloy is more pronounced and higher at each time interval as compared to the Al-2Cu alloy. Binary alloy shows peak ageing response after 10 hours with a hardness value of 1260 ± 11 MPa, while hardness of quaternary Al-2Cu-0.1Nb-0.15Zr alloy peaked at 5 hours with the hardness value of 1500 ± 8 MPa, which is roughly 19% higher than the binary alloy. Figure 2(a) shows a STEM HAADF contrast image near [001] zone axis that reveals that most of the plate precipitates have heterogeneously nucleated (having three variants) on the pre-existing spherical Al_3_Zr ordered precipitates. In few cases, independent nucleation of the plate could also be observed. The nature of the heterogeneous nucleation is further confirmed by high-resolution STEM micrograph taken near [001] zone axis (Fig. 2(b)). To determine the structure of these precipitates, diffraction patterns were obtained along [001] zone axis from the peak aged sample, as shown in Fig. 2(c). The diffraction pattern contains both superlattices ordered spots corresponding to L1_2_ spherical precipitates and streaks along < 010 > directions arising from plate-shaped precipitates. Examination along 020 direction in the reciprocal space reveals existence of two distinct patterns. This is highlighted in the cut strips from the overall pattern marked as (i) and (ii). S5 shows the unit cell structures and the lattice parameters for θ″ (Gerold Model), θ′ and L1_2_ ordered precipitates. In S6I and S6II, the experimentally obtained patterns (a) are compared with the simulated patterns (b). Indexed simulated patterns of individual θ′/θ″ variants overlapped with [001]_Al_ pattern are also included. The pattern in Fig. 2(c,i) contains streaks with maxima at 1/4 020_Al_, 1/2 020_Al_ and 3/4 020_Al_ positions that are characteristics of θ″ precipitates (indexed as $00\bar{1}$_θ″_, $00\bar{2}$_θ″_ and $00\bar{3}$_θ″_ respectively). It is to be noted that that ${010}_{{\rm{L}}{1}_{2}}$ superlattice spot of L1_2_ precipitates overlaps with the $00\bar{2}$_θ″_ (See S6I for other overlapping spots). These θ″ precipitates are always associated with heterogeneously nucleated plates. These plate-shaped precipitates are much finer having length of 26 ± 18 nm and have much higher number density as compared to the one that is not heterogeneously nucleated. The orientation relationship between fcc α-Al matrix, L1_2_ Al_3_Zr and θ″ precipitates can be written as

$$\begin{array}{c}{[{\bf{001}}]}_{{\boldsymbol{Al}}}\parallel {[{\bf{001}}]}_{{{\boldsymbol{\theta }}}^{^{\prime\prime} }}\parallel {\{{\bf{001}}\}}_{{\boldsymbol{L}}{{\bf{1}}}_{{\bf{2}}}}\\ {({\bf{100}})}_{{\boldsymbol{Al}}}\parallel {({\bf{100}})}_{{{\boldsymbol{\theta }}}^{^{\prime\prime} }}\parallel {\langle {\bf{100}}\rangle }_{{\boldsymbol{L}}{{\bf{1}}}_{{\bf{2}}}}\end{array}$$

APT measurements (Fig. 2(d)) show the plate-shaped precipitates (orange color) to be copper rich and nucleated on the Zr-rich spherical precipitates (purple color). The composition of the copper rich and Zr-rich precipitates is measured to be Al-13.3Cu-0.2Nb-0.57Zr and Al-1.02Cu-0.8Nb-23.24Zr respectively (Figure S7). The copper content in the plate-shaped precipitates also suggests that these are θ″ precipitates having composition closer to the stoichiometric Al_3_Cu. The θ′ precipitates, observed occasionally, are fewer in number and the microstructure is dominated by θ″ precipitates.

**Figure 3:**
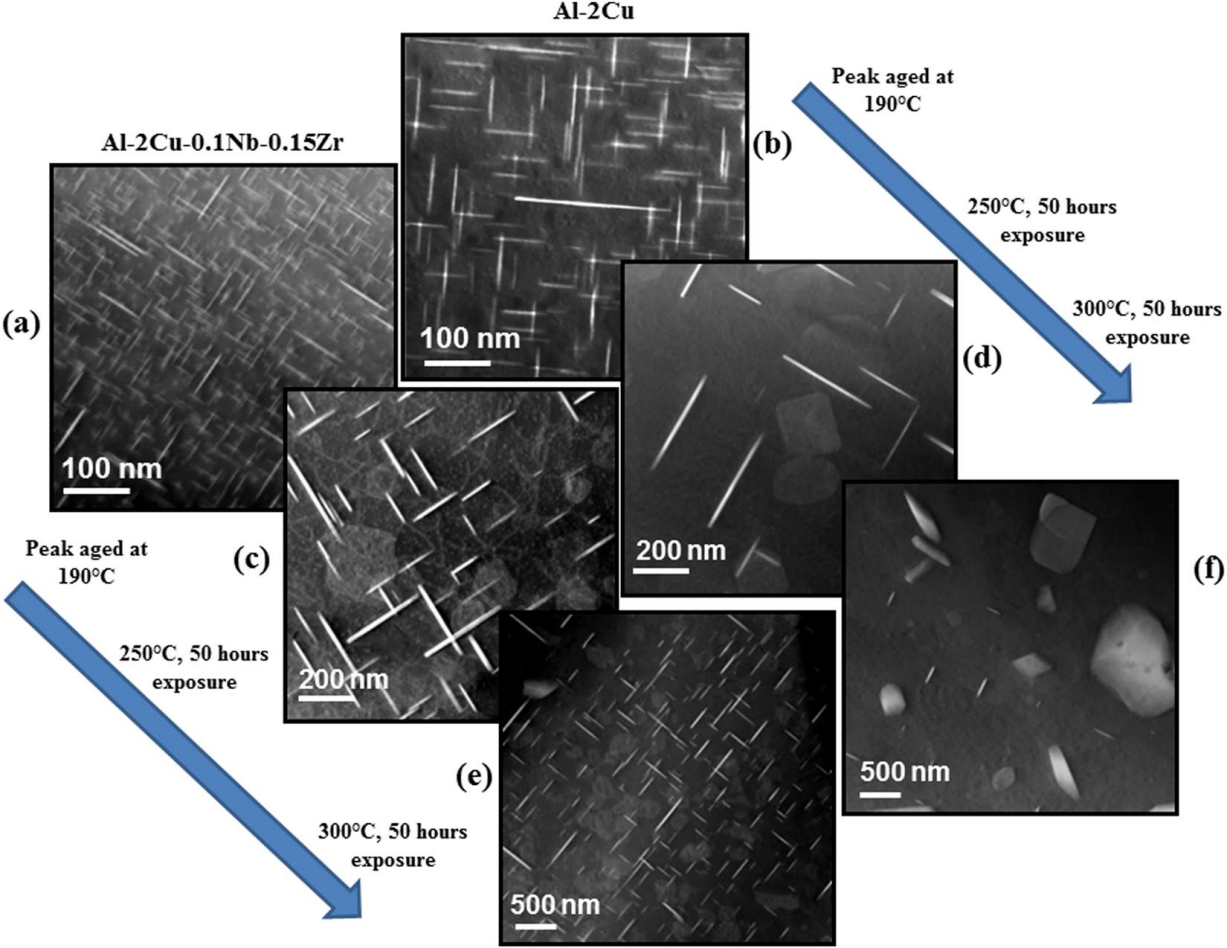
Microstructural comparison of Al-2Cu-0.1Nb-0.15Zr and Al-2Cu alloys. Al-2Cu-0.1Nb-0.15Zr alloy after (a) final step agi**n**g at 190 °C for 5 hours, (**c**) 50 hours exposure at 250 °C and (**e**) 50 hours exposure at 300 °C. Al-2Cu alloy after (**b**) peak aging at 190 °C for 10 hours, (**d**) 50 hours exposure at 250 °C and (**f**) 50 hours exposure at 300 °C.

Figure 3(a) and (b) shows the peak aged microstructure of the Zr-containing quaternary alloy and the binary Al-2Cu alloy near [001] zone axis respectively. It is evident that the quaternary alloy has much finer distribution of copper rich plates with higher number density than the binary alloy. The average length and the number density of the plates are 113 ± 43 nm and 0.9 × 10^21^ m^−3^ respectively for peak aged binary Al-2Cu alloy, while for Zr-containing quaternary alloy the maximum size observed is 90 nm with average of 31 ± 28 nm and the number density is 18 × 10^21^ m^−3^.

The stability of the peak aged microstructure for both the alloys after 50 hours of exposure at 250 °C and 300 °C is shown in Fig. 3(c–f) respectively. It is evident that the reduction in number density (Figure S8a) and increase in the length of the plates (Figure S8b) is more pronounced in the case of binary Al-2Cu alloy. After exposure to 300 °C for similar time (50 hours), the precipitates remain plate shaped in quaternary alloy, while in binary alloy, most of them transform to coarse equilibrium θ precipitates in the α-Al matrix. The number density of copper rich plates decreases with time for both the alloys. However, the effect is more pronounced for Al-2Cu alloy as compared to Al-2Cu-0.1Nb-0.15Zr alloy. This decrease in the number density is reflected in a sharp decrease in the hardness value (Figure S8c) for the binary alloys.

### Phase field simulation

To understand this further, we have carried out phase field simulations of microstructural evolution in model quaternary and binary alloy systems^20^. In the simulations, the interfacial energies between the coexisting Al_3_Zr, θ″ and the Al-rich matrix phases are assumed to be similar. The misfit between Al_3_Zr and the Al-rich matrix phase is positive and dilatational in nature (estimated to be + 0.007 from the experimental diffraction data). The coherent θ″ precipitates appear in the form of plates with the broad faces having a small positive lattice mismatch (~ + 0.002) and the edges having a large and negative misfit (~ −0.05) with respect to the matrix phase. Further, the atomic mobility of Zr is assumed smaller than that of Cu at the ageing temperature. The quaternary alloy system is approximated with a simpler ternary alloy system containing three atomic species A, B and C. B and C denote the principal alloying elements in the system. At a particular aging temperature, the alloy exhibits two ordered phases (denoted as $\,\beta$ and $\,\gamma$) embedded in the disordered matrix $\alpha$. Let ${c}_{i}({\boldsymbol{r}},{\rm{\tau }})\,(i=A,B,C)$ represent the scaled composition of each species as a function of position, r and time, ${\rm{\tau }}.$ so that $\sum _{i=A,B,C}{c}_{i}(r,{\rm{\tau }})=1,\,{c}_{i}(r,{\rm{\tau }})\ge 0$. An order parameter field $\varphi (r,{\rm{\tau }})$ is used to describe the ordered $\beta$ phase ($A{l}_{3}Zr$ phase). The ordered $\gamma$ phase has a tetragonal symmetry (θ″ phase) whose orientational variants are represented by three non-conserved order parameter fields $\,{\eta }_{i}({\boldsymbol{r}},{\rm{\tau }})(i=1,2,3)$.

The total free energy,$\,F$, of the system is expressed as a sum of its chemical $\,({F}_{c})$ and elastic $({F}_{e})$ parts:

$F={F}_{c}+{F}_{e}$. The chemical free energy,$\,{F}_{c}$, of the system as a function of the field variables is given as

1 $${F}_{c}={N}_{v}\mathop{\int }\limits_{V}({f}_{0}({c}_{B},{c}_{C},\varphi ,{\eta }_{1},{\eta }_{2},{\eta }_{3})+\sum _{i=A,B,C}{\kappa }_{i}{(\nabla {c}_{i})}^{2}+{\kappa }_{\varphi }{(\nabla \varphi )}^{2}+\sum _{j=1,2,3}{\kappa }_{{\eta }_{j}}{(\nabla {\eta }_{j})}^{2})dV$$

where ${N}_{v}$ is the number of molecules per unit volume, ${f}_{0}({c}_{B},{c}_{C},\varphi ,{\eta }_{1},{\eta }_{2,}{\eta }_{3})$ is the bulk free energy density, ${\kappa }_{i},\,{\kappa }_{\varphi }\,$and ${\kappa }_{{\eta }_{j}}\,\,$are the gradient energy coefficients associated with the gradients in composition and order parameters respectively. Equation 1 represents the chemical energy of the system, which includes contributions from bulk free energy density arising from local chemical interactions, gradient energy contributions arising from gradients in composition fields $({{\rm{c}}}_{{\rm{B}}}({\bf{r}},{\rm{\tau }}),{c}_{{\rm{C}}}({\bf{r}},{\rm{\tau }}))$ and long range order parameter fields ($\varphi ({\bf{r}},{\rm{\tau }}),{{\rm{\eta }}}_{{\rm{i}}}({\bf{r}},{\rm{\tau }})\,({\rm{i}}=1,2,3)),$ which are non-zero around the interfaces^20^. The gradient energy terms contribute to the excess energy associated with the interfaces. The bulk free energy density is typically expressed as a polynomial expansion of the composition fields and the order parameter fields using a Landau-type expansion given in equation 2. The Landau-type polynomial accounting for the bulk free energy density of the model alloy system is given as:

2 $$\begin{array}{c}{f}_{0}={P}_{1}{c}_{A}^{2}{c}_{B}^{2}+{P}_{2}{c}_{B}^{2}{c}_{C}^{2}+{P}_{3}{c}_{A}^{2}{c}_{C}^{2}+Q{c}_{A}^{2}{c}_{B}^{2}{c}_{C}^{2}\,+{R}_{1}{({c}_{C}-{\eta }_{1}^{2}-{\eta }_{2}^{2}-{\eta }_{3}^{2})}^{2}+{R}_{2}{({c}_{B}-{\varphi }^{2})}^{2}+{S}_{1}{\varphi }^{2}({\eta }_{1}^{2}+{\eta }_{2}^{2}+{\eta }_{3}^{2})\,+{S}_{2}({\eta }_{1}^{2}{\eta }_{2}^{2}+{\eta }_{2}^{2}{\eta }_{3}^{2}\,+\,{\eta }_{1}^{2}{\eta }_{3}^{2}),\end{array}$$

where the coefficients ${P}_{1},{P}_{2},{P}_{3},\,Q,\,{R}_{1},\,{R}_{2},{S}_{1}\,and\,{S}_{2}$ are the phenomenological constants in the model^21^. The coefficients are adjusted to describe the coexistence of a disordered matrix phase and two ordered phases at a given temperature. The coefficients can be obtained from thermodynamic data for the relevant systems. Koyama developed a similar expression for the free energy density to describe a mixture of ${\rm{\gamma }}\,({\rm{A}}1),\,{\rm{\gamma }}\text{'}({\rm{L}}{1}_{2})$ and ${\rm{\gamma }}\text{'}\text{'}({{\rm{DO}}}_{22})$ phases in Ni-base superalloys^22^.

The bulk free energy function has eight global minima at

3 $$({c}_{B},{c}_{C},\varphi ,{\eta }_{1},{\eta }_{2},{\eta }_{3})=(0,0,0,0,0,0),(1,0,1,0,0,0),\,(0,1,0,\pm 1,0,0),\,(0,1,0,0,\pm 1,0)\,{and}\,(0,1,0,0,0,\pm 1)$$

The first minimum corresponds to equilibrium $\alpha$ phase (Al-matrix), the second one corresponds to ordered $\beta$ precipitates (L1_2_ Al_3_Zr) and the remaining ones correspond to the six variants of tetragonal $\gamma$ phase (θ″). The free energy surface as a function of composition variables, ${c}_{B}$ and ${c}_{C},\,\,$is shown in S9. The well associated with ${c}_{C}=1$ corresponds to one of the orientational variants of the ordered $\gamma$ phase. Since we have performed two dimensional simulations we consider two ordered orientation variants of the tetragonal $\gamma$ phase and set ${\eta }_{3}=0.$

We use homogeneous modulus approximation to obtain the elastic strain energy of the system. The elastic strain energy of an elastically homogenous coherent microstructure is evaluated by using Khachaturyan’s microelasticity theory^23^:

4 $${F}_{e}=\int {\sigma }_{ij}^{el}({\boldsymbol{r}}){{\epsilon }}_{ij}^{el}({\boldsymbol{r}})dV={\lambda }_{ijkl}({{\epsilon }}_{ij}^{^{\prime} }({\boldsymbol{r}})+{E}_{ij}-{{\epsilon }}_{ij}^{0}({\boldsymbol{r}}))({{\epsilon }}_{kl}^{^{\prime} }({\boldsymbol{r}})+{E}_{kl}-{{\epsilon }}_{kl}^{0}({\boldsymbol{r}}))$$

where ${\sigma }_{ij}^{el}({\boldsymbol{r}}),\,{{\epsilon }}_{ij}^{el}({\boldsymbol{r}}),\,{\lambda }_{ijkl},\,{{\epsilon }}_{ij}^{^{\prime} }({\boldsymbol{r}}),\,{{\epsilon }}_{ij}^{0}({\boldsymbol{r}})\,{\rm{and}}\,{E}_{ij}$ are stress, elastic strain, elastic stiffness tensor, eigenstrain, periodic strain and homogenous strain respectively. The total eigenstrain field ${\epsilon }^{0}$, is the sum of three eigenstrain fields ${{\boldsymbol{\epsilon }}}^{0}(\varphi )$, ${{\boldsymbol{\epsilon }}}^{0}({\eta }_{1})\,$ and ${{\boldsymbol{\epsilon }}}^{0}({\eta }_{2})$ and is expressed as

5 $${{\boldsymbol{\epsilon }}}_{ij}^{0}=\sum _{p=0}^{2}{\theta }_{p}{{\boldsymbol{\epsilon }}}_{ij}^{(p)\,}$$

where ${\theta }_{0}=\varphi$, ${\theta }_{i}={\eta }_{i}^{2}\,(i=1,2)\,\,$and ${{\boldsymbol{\epsilon }}}_{ij}^{(p)\,}\,$represents the position independent part of the eigenstrain field associated with ${\theta }_{p}.$

The tetragonal θ″ phase has anisotropic, crystallographically equivalent misfit with the $\alpha$ matrix. The precipitate-matrix misfit tensor is anisotropic and is expressed as $${{\epsilon }}^{\ast }=(\begin{array}{cc}{{\epsilon }}_{xx} & 0\\ 0 & {{\epsilon }}_{yy}\end{array})={{\epsilon }}_{p}(\begin{array}{cc}1 & 0\\ 0 & t\end{array}),$$ where ${{\epsilon }}_{p}$ denotes the characteristic misfit strain and $t={{\epsilon }}_{yy}/{{\epsilon }}_{xx}$ is defined as the misfit anisotropy parameter. For dilatational misfit,$\,t=1$. The eigenstrain fields in the model are ${{\boldsymbol{\epsilon }}}_{ij}^{(0)\,}={{\epsilon }}_{\beta }{\delta }_{ij}$,$$\,{{\boldsymbol{\epsilon }}}_{ij}^{(1)\,}={{\epsilon }}_{\eta }(\begin{array}{c}1\,0\\ \,0\,t\end{array})$$, and $${{\boldsymbol{\epsilon }}}_{ij}^{(2)\,}={{\epsilon }}_{\eta }(\begin{array}{c}t\,0\\ \,0\,1\end{array})$$. ${{\epsilon }}_{\beta }$ is the lattice parameter difference between the ordered $\beta \,(L{1}_{2})$ phase and the disordered matrix $(\alpha )$ while ${{\epsilon }}_{\eta }$ denotes the strength of misfit between ordered $\theta ^{\prime\prime}$ and $\alpha$ ^21^.

The spatiotemporal evolution of microstructure is studied by solving Cahn-Hilliard equation for the composition fields and Allen-Cahn equation for the order parameter fields:

6 $$\frac{\delta {c}_{i}}{\delta {\rm{\tau }}}={M}_{i}{\nabla }^{2}\frac{\delta F}{\delta {c}_{i}}\,+{\xi }_{{c}_{i}},\,(i=B,C)$$

7 $$\frac{\partial {\theta }_{i}}{\partial {\rm{\tau }}}=-L\frac{\delta F}{\delta {\theta }_{i}}+{\xi }_{{\theta }_{i}},({\theta }_{0}=\varphi ,\,{\theta }_{1}={\eta }_{1},{\theta }_{2}={\eta }_{2}),$$

where, ${\xi }_{{c}_{i}}\,\,$ and ${\xi }_{{\theta }_{i}}$ are the Langevin force terms added to mimic the nucleation process^20–22^.

The simulations are carried out in a two dimensional system of size $\,1024\times 1024$. All the parameters in the simulations are non dimensionalized using characteristic length, time and energy parameters. The gradient energy coefficients are adjusted so that the interfacial energies between the coexisting phases are nearly equal. ${{\epsilon }}_{\beta }$ is chosen to be 0.007 to describe a positive dilatational misfit associated with $\beta$ phase; ${{\epsilon }}_{\eta }$ and $t$ are chosen to be −0.05 and −0.04 such that the broad faces of the plates of ordered $\gamma$ has a small positive misfit with the matrix and the rims are associated with a large negative misfit. We use semi-implicit Fourier spectral method to solve the governing equations with periodic boundary conditions.

**Figure 4:**
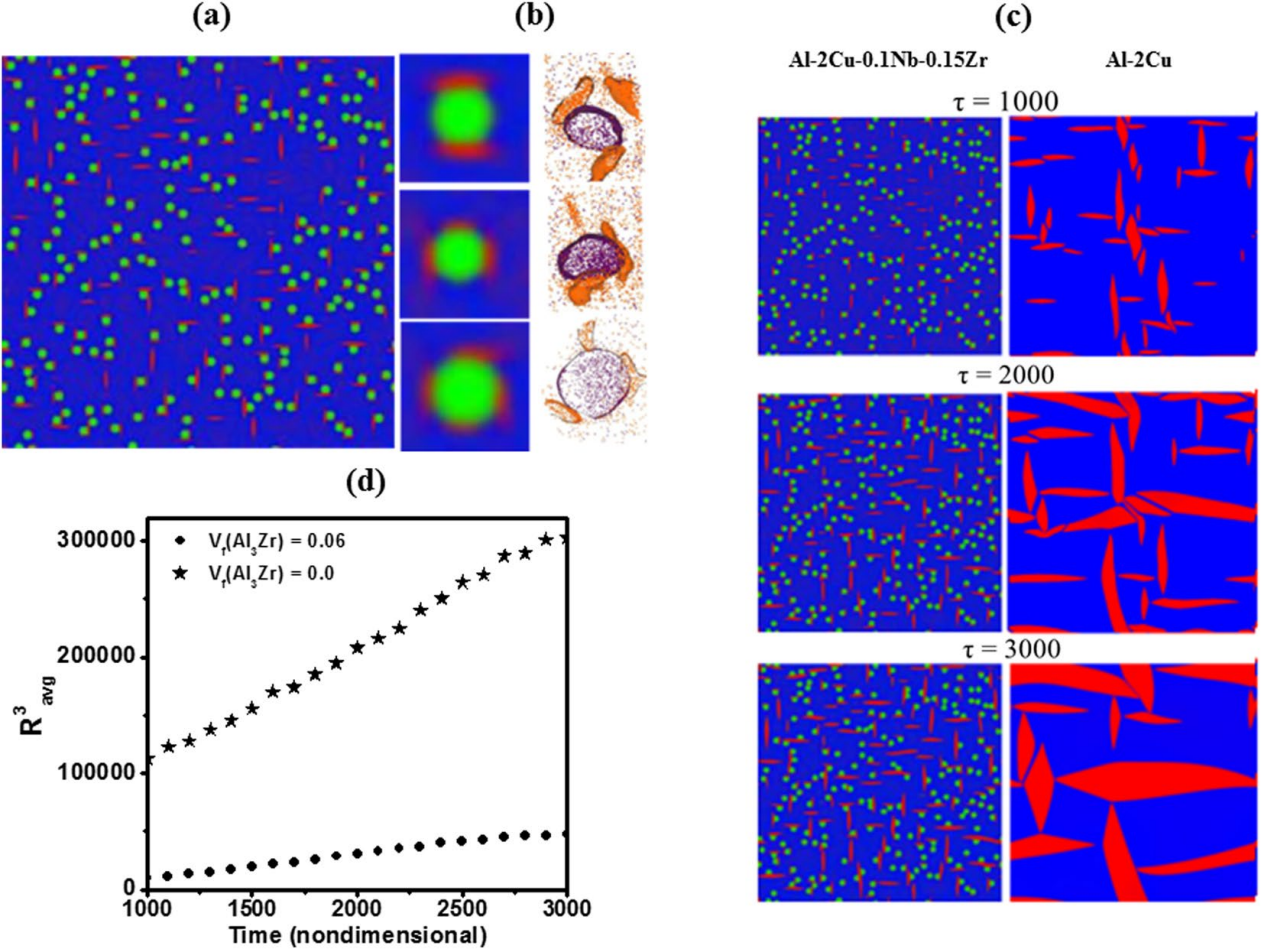
(**a**) Heterogeneous $\mathrm{nucleation}\,\mathrm{of}\,$θ″ precipitates on pre-existing Al_3_Zr precipitates obtained from phase field simulations, (b) examples of compact strain accommodating patterns where 2, 3, 4 crystallographic variants of θ″ enclose Al_3_Zr precipitates with experimental APT reconstructions, (**c**) comparison of time snapshots of evolution of θ″ precipitates in the presence and absence of Al_3_Zr precipitates obtained from phase field simulations and (**d**) rate of coarsening of plate shaped precipitates (determined using Hoshen-Kopelman algorithm) compared for the two systems.

Figure 4(a) shows the simulated phase field microstructure for the quaternary alloy after the final ageing at 190 °C for 1000 s. It resembles the experimentally obtained microstructure that shows plate-shaped θ″ precipitates primarily forming on the surfaces of Al_3_Zr precipitates, although we do observe a few independent nuclei of θ′ in the matrix. Figure 4(b) shows examples of compact strain accommodating patterns, where 2, 3, 4 crystallographic variants of θ″ enclose Al_3_Zr precipitates and their comparison with APT reconstructions. The formation of θ″ on the surfaces of pre-existing Al_3_Zr precipitates gives rise to compact (often sandwich-like) morphological features, where the central Al_3_Zr precipitate is surrounded by plates of θ″. Figure 4(c) shows the comparison of time snapshots of evolution of θ″ precipitates in the presence and absence of spherical Al_3_Zr precipitates. It can be established that in the compact morphological microstructure, the θ″ precipitates grow at a significant slower rate compared to the homogeneously formed θ″ precipitates in binary Al-2Cu alloy.

## Discussion

The slower growth kinetics for the quaternary Al-Cu-Nb-Zr alloy as compared to the binary Al-Cu alloy can be discussed on the basis of two factors; additional elastic strain energy minimization and Nb (and Zr) solute-vacancy binding energy.

The formation of compact precipitates is attributed to the minimization of elastic strain energy. Al_3_Zr precipitates provide heterogeneous nucleation sites to θ″ precipitates. The broad faces of θ″ precipitates enclose the Al_3_Zr precipitates leading to formation of “compact” precipitate morphology. Formation of such compact precipitates leads to a significant reduction in coherency strain, thereby reducing the elastic strain energy. For example, in a sandwich-like morphology where two θ″ plates of same crystallographic orientation enclose an Al_3_Zr precipitate, the effective misfit strain reduces to + 0.003. Further, the overlapping of strain and diffusion fields when Al_3_Zr is bounded on all the sides with θ″ variants restricts the growth of the variants. Similar compact/ composite morphologies with excellent thermal stability have been reported in INCONEL 718, Al-Li-Zr, and Al-Li-Sc-Zr alloy systems^25–27^. Moreover, the compact precipitates, formed because of strain accommodation, exhibit a uniform (‘monodispersed’) size distribution resulting in significantly delayed coarsening. We should also note that the presence of prior Al_3_Zr precipitates is the key to the formation of compact morphology – they contribute to the reduction of misfit strain as well as retention of compact morphology with a uniform size distribution. Therefore, the sluggish diffusion of Zr through the matrix also significantly retards the growth and coarsening of the compact morphology. On the other hand, the elongation of θ″ plates along low misfit directions and their thickening happens far more rapidly in the absence of Al_3_Zr precipitates. The rapid growth of isolated θ″ plates may lead to loss of coherency along the edges and transformation to semi-coherent θ′ plates. Through the phase-field simulations, we demonstrate that growth kinetics of θ″ plates is indeed affected by the presence of coherent Al_3_Zr precipitates. The simulated coarsening rate for the θ″ plates is significantly lower in the microstructure containing Al_3_Zr precipitates (Fig. 4(d)). The average size of θ″ plates at each time step is calculated using a cluster counting algorithm developed by Hoshen and Kopelman^24^. The total number of particles (number of red clusters) and the total area occupied by the clusters is determined at each time step from which we compute an average cluster size. To obtain the coarsening rate, counting is started at ${\rm{\tau }}=1000$ (approximately one million simulation steps) where coarsening is dominant and supersaturation in the matrix has significantly reduced. We continue till three million simulation steps (${\rm{\tau }}=3000)$ where the simulation box contains at least twenty interfaces to ensure good statistics.

One additional factor to be considered is Nb and Zr solute – vacancy interaction in α-Al matrix. Atom Probe data shows some amount of Nb and Zr still remains in the α-Al matrix. In Al alloys, Nb and Zr atoms exhibits very high value of solute vacancy binding energy of the order 0.18 ± 0.02 eV^28^ and 0.24 ± 0.02 eV^29^ respectively as compared to Cu atoms (0.0 ± 0.12 eV)^30^. Since the diffusion of solute happens through vacancy migration, higher Nb and Zr solute-vacancy binding energy slows down the diffusion of Cu atoms in the matrix. Hence, the quaternary alloy coarsens at a slower rate than the binary Al-Cu alloy.

### Mechanical properties

**Figure 5:**
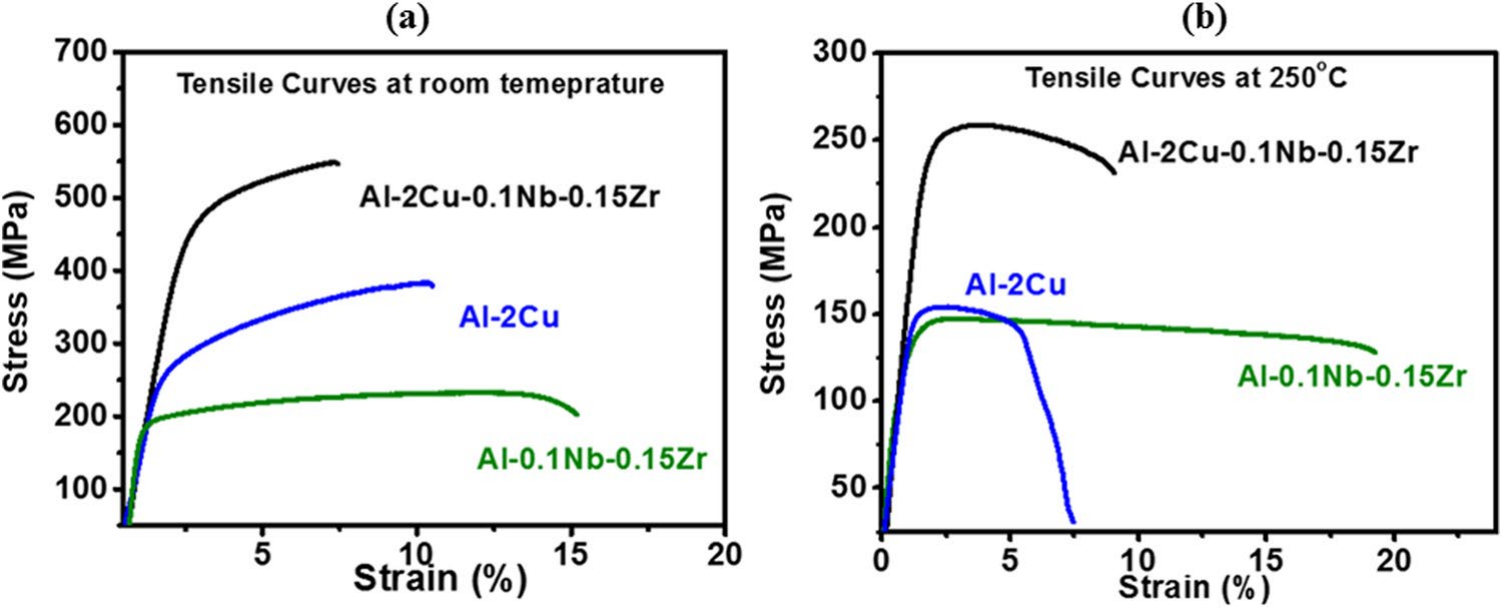
(**a**) Tensile test curves for Al-2Cu, Al-0.1Nb-0.15Zr and Al-2Cu-0.1Nb-0.15Zr alloys (peak aged at 190 °C) at room temperature and (**b**) at 250 °C.

Tensile properties were measured for quaternary Al-2Cu-0.1Nb-0.15Zr, ternary Al-0.1Nb-0.15Zr and binary Al-Cu alloys (peak aged at 190 °C) at room temperature and at 250 °C (Fig. 5). The 0.2% proof stress and UTS for quaternary alloy at room temperature were measured to be 460 ± 18 MPa and 540 ± 20 MPa (with 6 ± 0.9% elongation). The strength values are significantly higher than both the ternary (180 ± 14 MPa and 230 ± 16 MPa with 14 ± 1.2% elongation) and the binary alloys (265 ± 16 MPa and 395 ± 22 MPa respectively with 12.5 ± 1.2% elongation). At 250 °C, the strength values for quaternary alloy were 250 ± 16 MPa and 260 ± 19 MPa (with 8.5 ± 0.7% elongation), much higher than the ternary (135 ± 13 MPa and 145 ± 15 MPa with 19 ± 1.4% elongation) and binary alloys (130 ± 14 MPa and 140 ± 17 MPa). The synergistic effect of both type of precipitates in quaternary alloy is more pronounced at high temperature.

In conclusion, we have demonstrated a concept for designing aluminium alloys through solid state precipitation reactions that utilise differences in temperature required for nucleation of different types of precipitates. An alloy based on Al-Cu-Nb-Zr was developed containing both L1_2_ ordered spherical Al_3_Zr precipitates that are stable at high temperature and copper rich plates that give extensive room temperature strength. It is shown through phase field simulation that the heterogeneous nucleation of θ″ plates surrounding the Al_3_Zr spherical precipitates leads to reduction of elastic energy and reduces the kinetics of their growth and coarsening. These contribute to stability at high temperature as well as significant strengthening at room temperature and at 250 °C.

## Methods

### Alloy preparation

Alloy ingots (10 grams) of nominal composition Al-2Cu-0.1Nb-0.15Zr, Al-0.1Nb-0.15Zr and Al-2Cu were prepared by melting the constituent elements using vacuum arc melting unit under argon atmosphere. The melted ingots were cast in the chilled copper mould in the form of 3 mm diameter rods using vacuum suction cast unit.

### Microstructural analysis

Localized composition distribution was determined using a field emission tipped electron probe micro analyzer (EPMA, JEOL make). Details of the microstructure, phase identification (through diffraction analysis) were obtained using a transmission electron microscope (TEM) equipped with field emission source (FEI F30) and analytical transmission electron microscope (JEOL 2000FX) having tungsten filament. Samples for the TEM analysis were prepared by mechanical polishing up to 40μm followed by formation of hole to prepare the samples electron transparent using Gatan PIPS operated at 5 keV at 4–4° with dual beam mode. To calculate the number density, volume fraction and precipitate size, foil thickness for the aged samples were measured using convergent beam technique and STEM HAADF micrographs taken from the same region. Experimental diffraction patterns were simulated in JEMS diffraction software with known lattice parameters. To obtain actual lattice misfit between matrix and precipitates, the experimental patterns were imported in the software and the simulated patterns were exactly overlapped on them with optimizing the lattice parameters.

The compositional partitioning of the solute atoms across the matrix and precipitates were determined by using atom probe tomography (APT). The samples for the APT analysis were prepared by a dual-beam focused ion beam (FIB) instrument (FEI Nova Nanolab 200^TM^) using a Ga ion beam. The thinning of the samples was carried out in multiple steps, starting with 30 kV ions finishing with 5 kV ions to reduce the surface damage caused by the higher energy ions. The final radius of curvature of the atom probe specimens was 50–80 nm. The APT experiments were carried out using a LEAP 3000X^TM^ local electrode atom probe system from Cameca Instruments Inc. All the atom probe experiments were carried out in the laser evaporation mode at a temperature of 40 K, with the evaporation rate varying from 0.5% to 0.7%. The laser pulse energy was set at 0.4 nJ while the pulse frequency was 160 kHz. Data analysis was performed using IVAS 3.6.8^TM^ software. Data analysis and proximity histograms (compositions vs distance from the interface) were generated using IVAS 3.6.8^TM^ software to get solute partitioning across the precipitate/matrix interface.

### Mechanical properties

For evaluating precipitation hardening response, the hardness of the samples was measured using Vickers microhardness tester (Zwick Roell ZHVI-A) with a load of 100 grams. Tensile tests were performed at room temperature and at 250 °C to determine mechanical properties of the alloys using Zwick Roell screw driven Universal Tensile Machine operated at a strain rate of 10^−3^ s^−1^. Round samples were machined according to ASTM standard E8/E8M-15A by keeping gauge length to diameter ratio 4 (gauge length 8 mm and diameter 2 mm).

## Electronic supplementary material

Supplementary

## References

[1] Bloch EA (1961). Dispersion-Strengthened Aluminium Alloys. Metall. Rev.

[2] Kelly A, Nicholson RB (1963). Precipitation Hardening. Prog. Mater. Sci..

[3] Gleiter H, Hornbogen E (1968). Precipitation hardening by coherent particles. Mater. Sci. Eng.

[4] Ardell AJ (1985). Precipitation hardening. Metall. Trans. A.

[5] Starke EA, Staley JT (1996). Application of modern aluminum alloys to aircraft. Prog. Aerosp. Sci..

[6] Chen CQ, Knott JF (1981). Effects of dispersoid particles on toughness of high-strength aluminium alloys. Met. Sci.

[7] Nie, J. F., Muddle, B. C. & Polmear, I. J. The effect of precipitate shape and orientation on dispersion strengthening in high strength aluminium alloys. in *Materials Science Forum* **217**, 1257–1262 (Trans Tech Publ, 1996).

[8] Marlaud T, Deschamps A, Bley F, Lefebvre W, Baroux B (2010). Influence of alloy composition and heat treatment on precipitate composition in Al–Zn–Mg–Cu alloys. Acta Mater..

[9] Chen K, Liu H, Zhang Z, Li S, Todd RI (2003). The improvement of constituent dissolution and mechanical properties of 7055 aluminum alloy by stepped heat treatments. J. Mater. Process. Technol..

[10] Paulisch MC (2015). The influence of heat treatments on the microstructure and the mechanical properties in commercial 7020 alloys. Mater. Sci. Eng. A.

[11] Reed-Hill, R. E. & Abbaschian, R. *Physical metallurgy principles*. (Brooks/Cole Engineering Division Monterey, Calif, USA, 1973).

[12] Radmilovic V (2011). Highly monodisperse core–shell particles created by solid-state reactions. Nat. Mater..

[13] Clouet E (2006). Complex precipitation pathways in multicomponent alloys. Nat. Mater..

[14] Voorhees PW (2006). Alloys: Scandium overtakes zirconium. Nat. Mater..

[15] Knipling KE, Dunand DC, Seidman DN (2006). Criteria for developing castable, creep-resistant aluminum-based alloys: A review. Z. Für Met.

[16] Davis, J. R. *et al*. Metals handbook: properties and selection: nonferrous alloys and special-purpose materials. (ASM International, 1990).

[17] Polmear, I. J. & Couper, M. J. Design and development of an experimental wrought aluminum alloy for use at elevated temperatures. *Metall. Trans. A* **19**, 1027–1035

[18] Reich L, Murayama M, Hono K (1998). Evolution of Ω phase in an Al–Cu–Mg–Ag alloy—a three-dimensional atom probe study. Acta Mater..

[19] Chuang MS, Tu GC (1995). The effect of Nb-addition on the L12 precipitates of rapidly-solidified Al-Cr-Zr alloy. Scr. Metall. Mater..

[20] Chen L-Q (2002). Phase-field models for microstructure evolution. Annu. Rev. Mater. Res.

[21] Bhattacharyya S, Abinandanan TA (2009). Evolution of multivariant microstructures with anisotropic misfit: A phase field study. Acta Mater.

[22] Koyama T (2010). Phase-field simulation of γ(Al)\+γ′(Ll_2_)+γ″(DO_22_) three-phase microstructure formation in Ni-base superalloys. Int. J. Mat. Res..

[23] Khachaturyan, A. G. *Theory of structural transformations in solids*. (Courier Corporation, 2013).

[24] Hoshen J, Kopelman R (1976). Percolation and cluster distribution. I. Cluster multiple labeling technique and critical concentration algorithm. Physical Review B.

[25] Cozar R, Pineau A (1973). Morphology of y′ and y″ precipitates and thermal stability of inconel 718 type alloys. Metall. Trans.

[26] He J, Han G, Fukuyama S, Yokogawa K (1998). Interfaces in a modified Inconel 718 with compact precipitates. Acta Mater..

[27] Gayle FW, Vander Sande JB (1984). “Composite” precipitates in an Al_3_Li_2_Zr alloy. Scr. Metall.

[28] Raman KS, Das ESD, Vasu KI (1970). Values of Solute-vacancy binding energy in aluminium matrix for Ag, Be, Ce, Dy, Fe, Li, Mn, Nb, Pt, Sb, Si, Y and Yb. Scripta Mater..

[29] Özbilen S, Flower HM (1989). Zirconium-vacancy binding and its influence on S′-precipitation in an Al-Cu-Mg alloy. Acta Metall..

[30] Wolverton C (2007). Solute-vacancy binding in aluminum. Acta Mater..

