# Supplementary material for: Enhancing elevated temperature strength of copper containing aluminium alloys by forming L12 Al3Zr precipitates and nucleating θ″ precipitates on them
Source: Sci Rep. 2017 Sep 11;7:11154. doi: 10.1038/s41598-017-11540-2 (PMC5593834; doi:10.1038/s41598-017-11540-2)
Supplement: Supplementary file 1 — Supplementary [file 41598_2017_11540_MOESM1_ESM.pdf]

Supplementary information

Enhancing elevated temperature strength of copper containing aluminium alloys by forming $L1_2$ Al_3Zr precipitates and nucleating θ" precipitates on them

Surendra Kumar Makineni[1,4,*], Sandeep Sugathan[2], Subhashish Meher[3], Rajarshi Banerjee[3], Saswata Bhattacharya[2], Subodh Kumar[1] and Kamanio Chattopadhyay[1]

[1]Indian Institute of Science, Department of Materials Engineering, Bangalore, 560012 India
[2]Indian Institute of Technology, Department of Material Science and Metallurgical Engineering, Hyderabad, 502285, India
[3]University of North Texas, Center for Advanced Research and Technology and Department of Materials Science and Engineering, Denton, TX-76203, USA
[4]Max-Planck-Institut für Eisenforschung, Department of Microstructure Physics and Alloy Design, Düsseldorf, 40237, Germany
*Corresponding Author: surendra.makineni@gmail.com

S1: Supplement 1

WDS elemental mapping for the chill cast quaternary alloy.

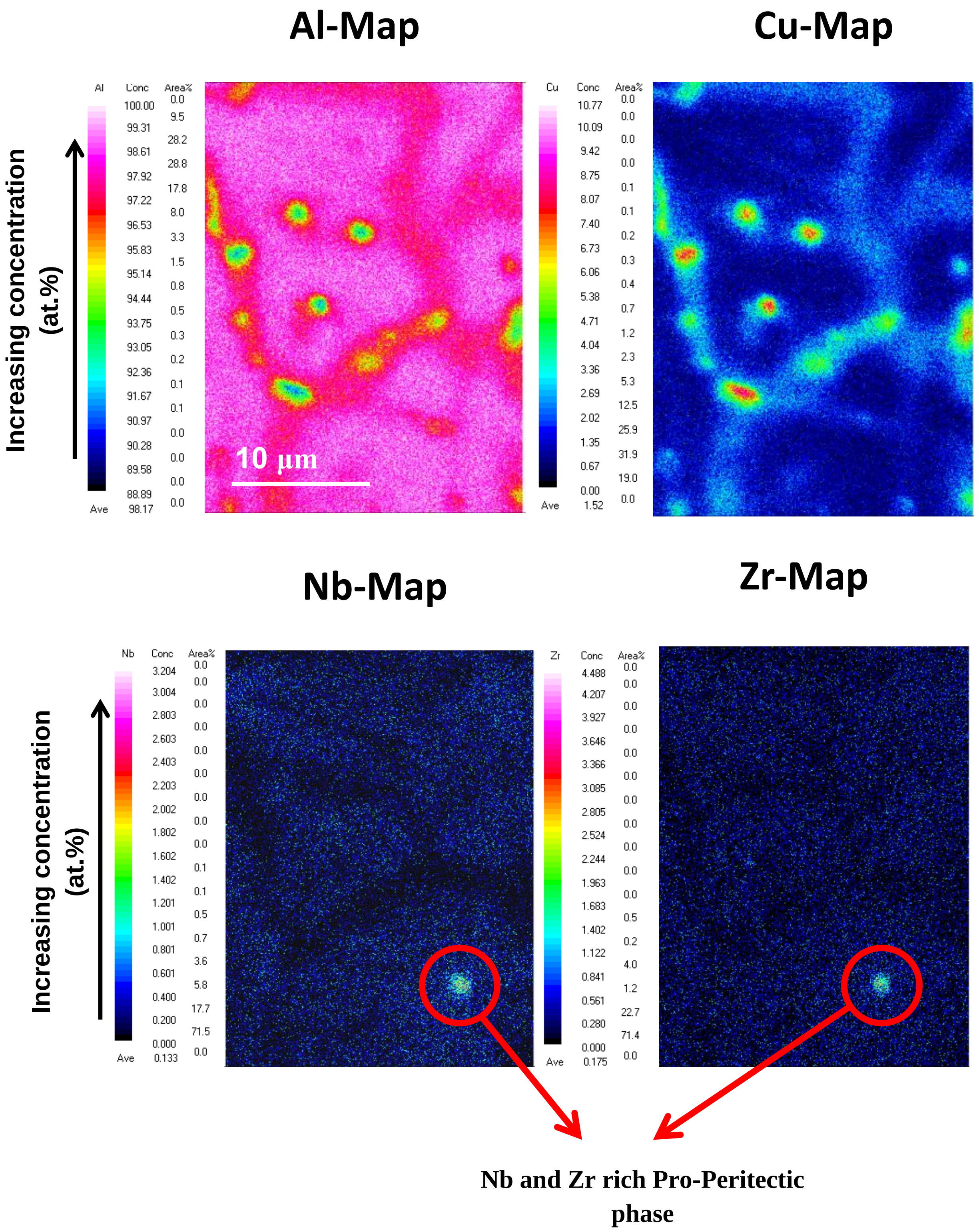

S2: Supplement 2

(a)

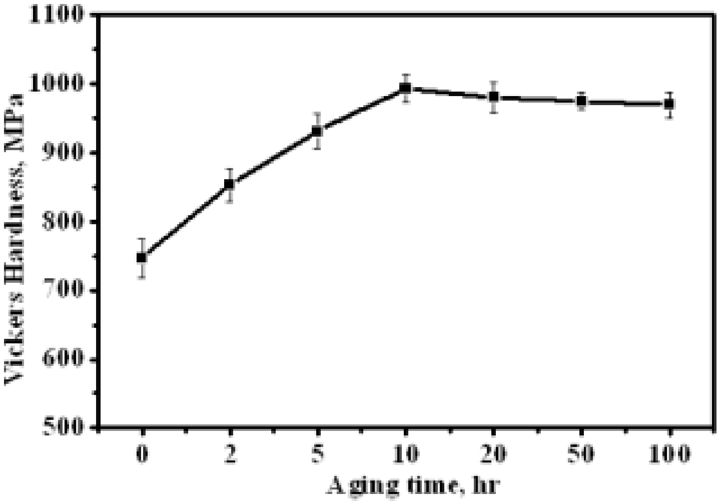

(b)

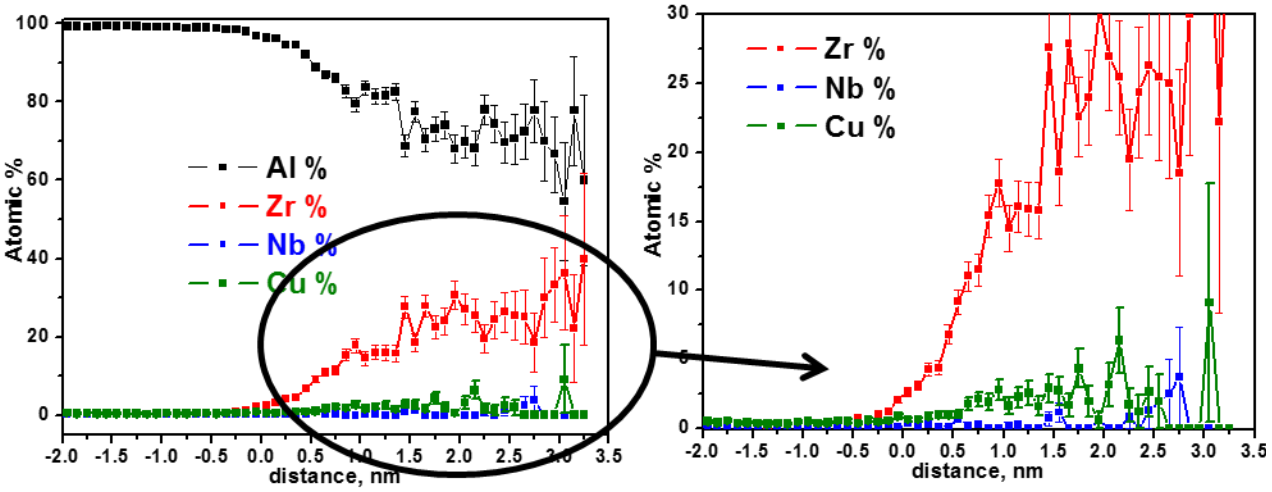

S2: (a) Hardness vs time plot for the chill cast alloy aged directly at 400°C (b) Proximity histogram across the spherical $L1_2$ precipitate/matrix interface using Atom Probe Tomography (APT)

S3: Supplement 3

WDS elemental mapping for the quaternary alloy after solutionising at 535°C for 30 minutes

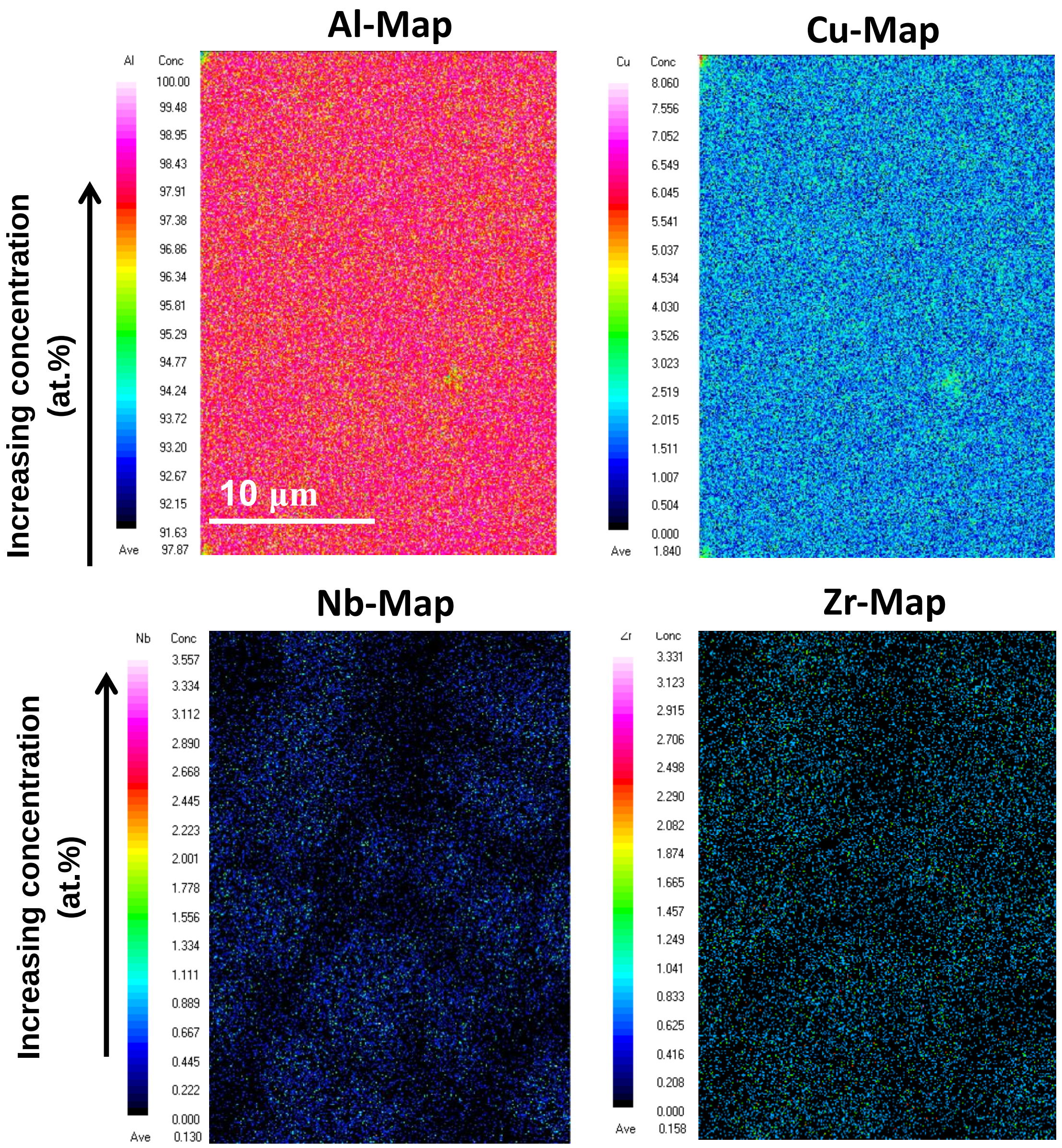

Please note that our heat treatment homogenises only the Cu atoms in the entire Al matrix and not the Nb/Zr atoms. The measured volume fraction of these cellular regions is around 80% and of intercellular region is 20%. Al_3Zr precipitates form on aging at 400°C, and Copper rich plate precipitates nucleated on Al_3Zr precipitates on aging at 190°C only in this cellular region.

S4: Supplement 4

(a)

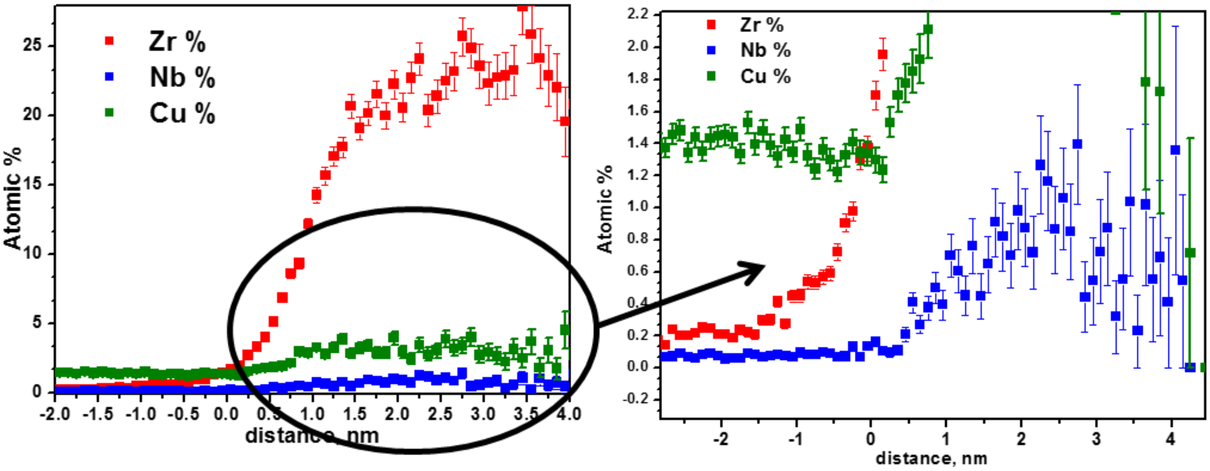

(b)

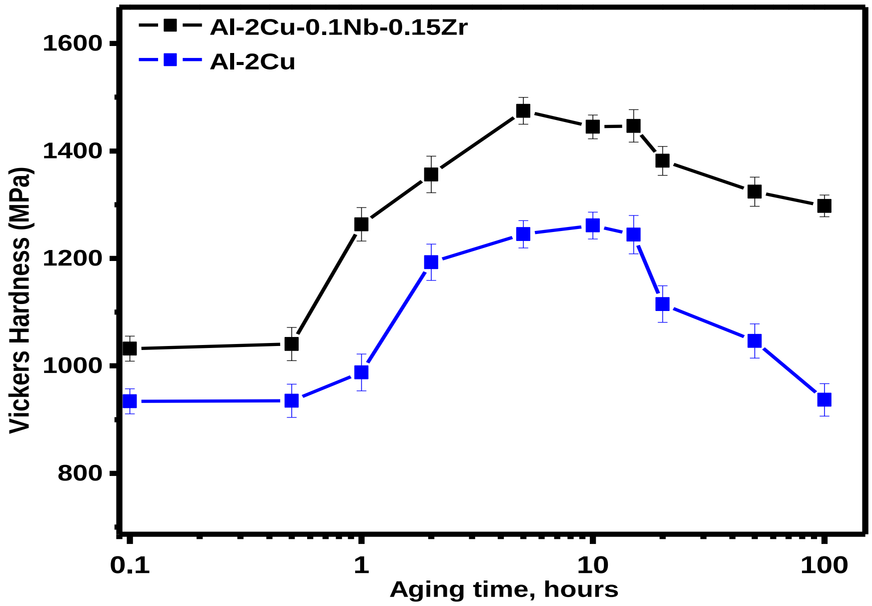

S4: (a) Proximity histogram across the spherical L1_2 precipitate/matrix interface for Al-2Cu-0.1Nb-0.15Zr alloy after solutionising for 30 minutes at 535°C (b) Hardness vs time plot for solutionised Al-2Cu-0.1Nb-0.15Zr and Al-2Cu alloys during aging at 190°C.

S5: Supplement 5

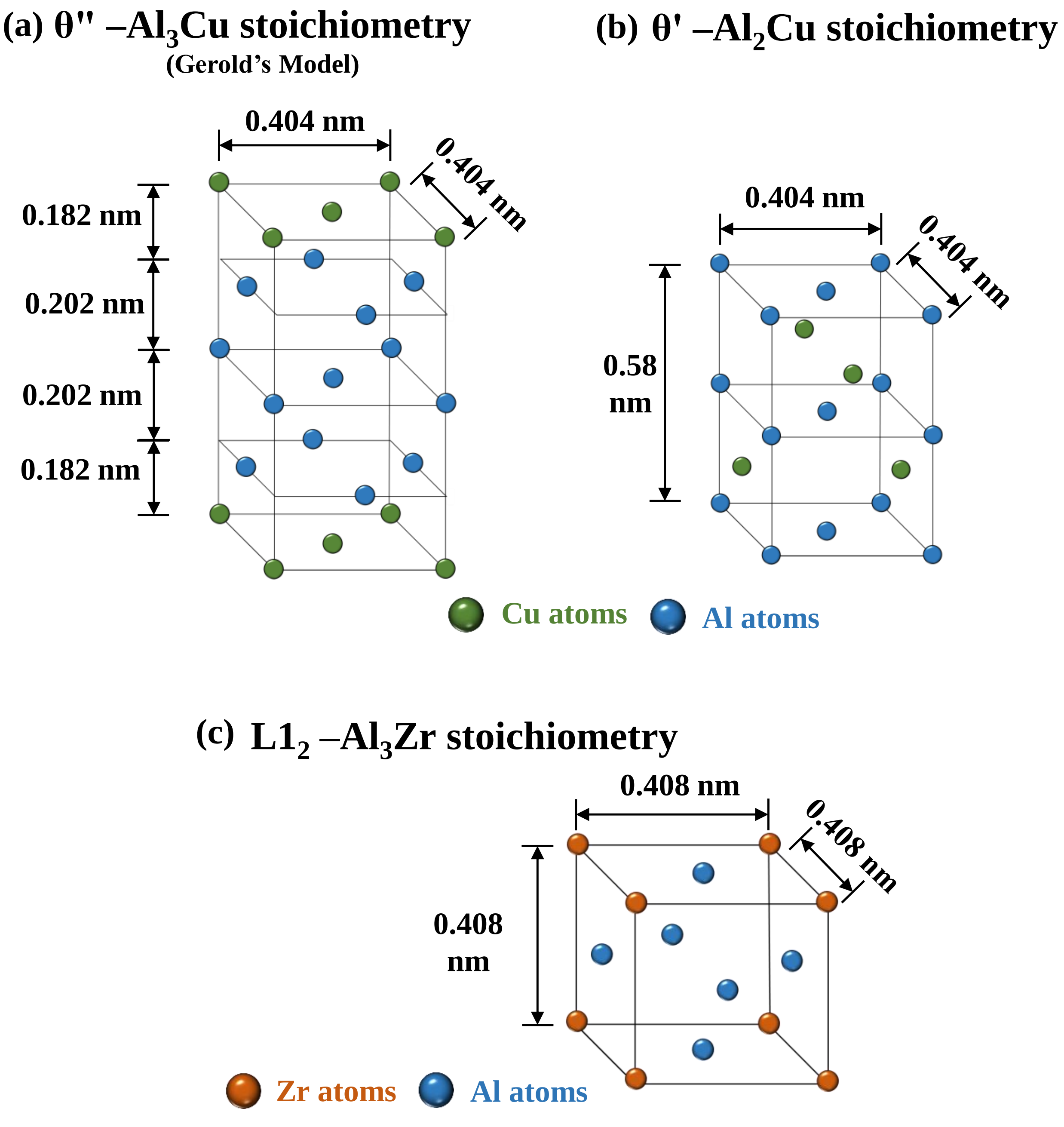

S6: Supplement 6

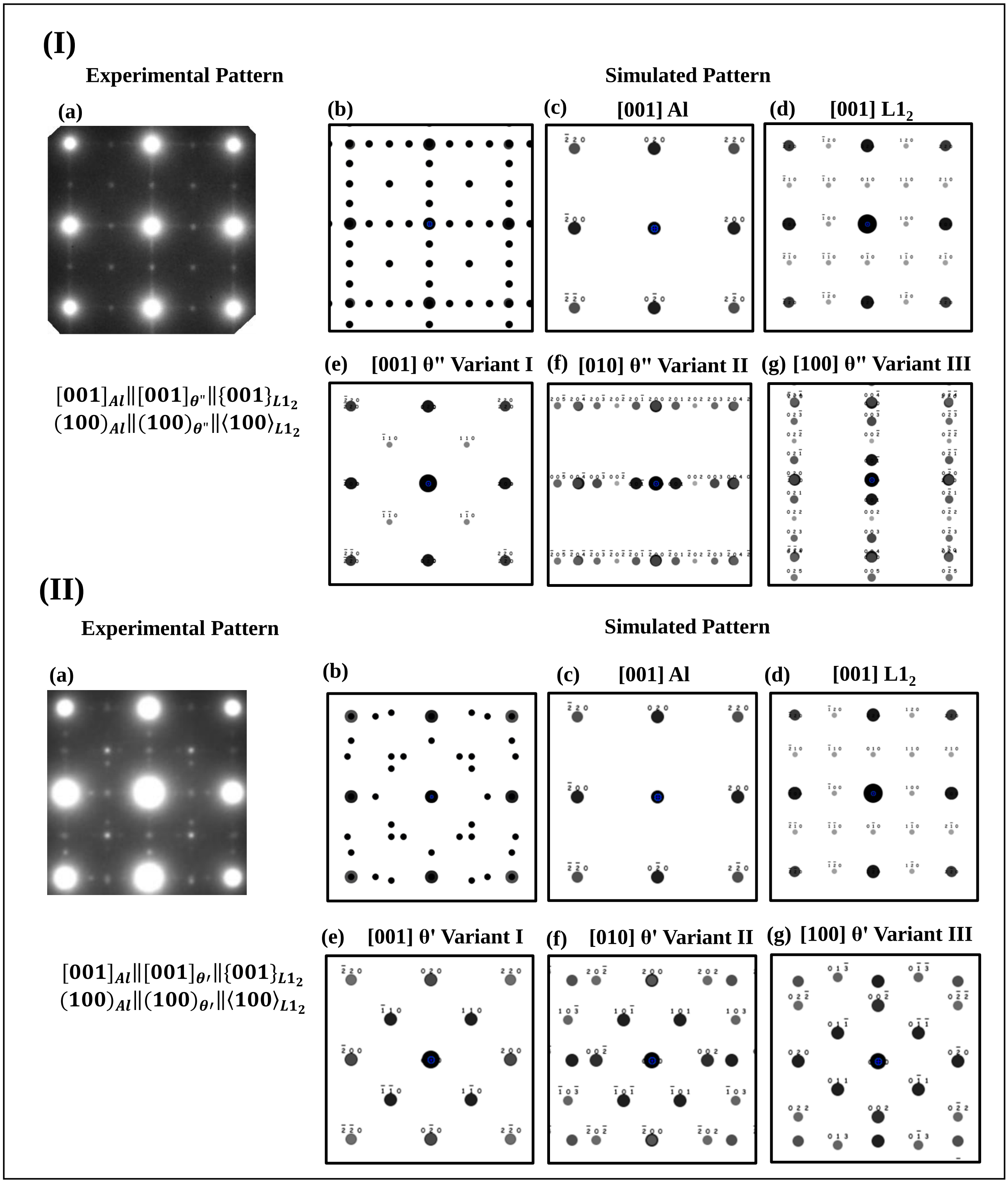

S7: Supplement 7

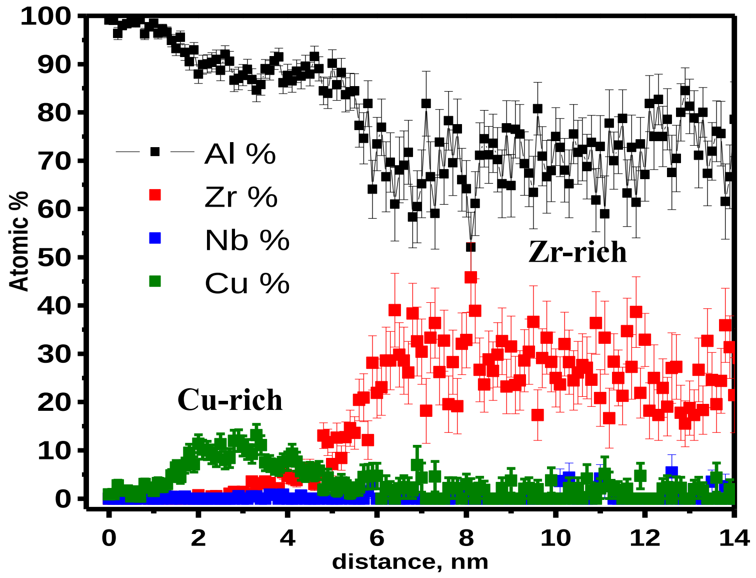

S7: Concentration profile across the matrix/θ"/L1_2 precipitate interfaces for quaternary alloy after peak aging at 190°C for 5 hours.

S8: Supplement 8

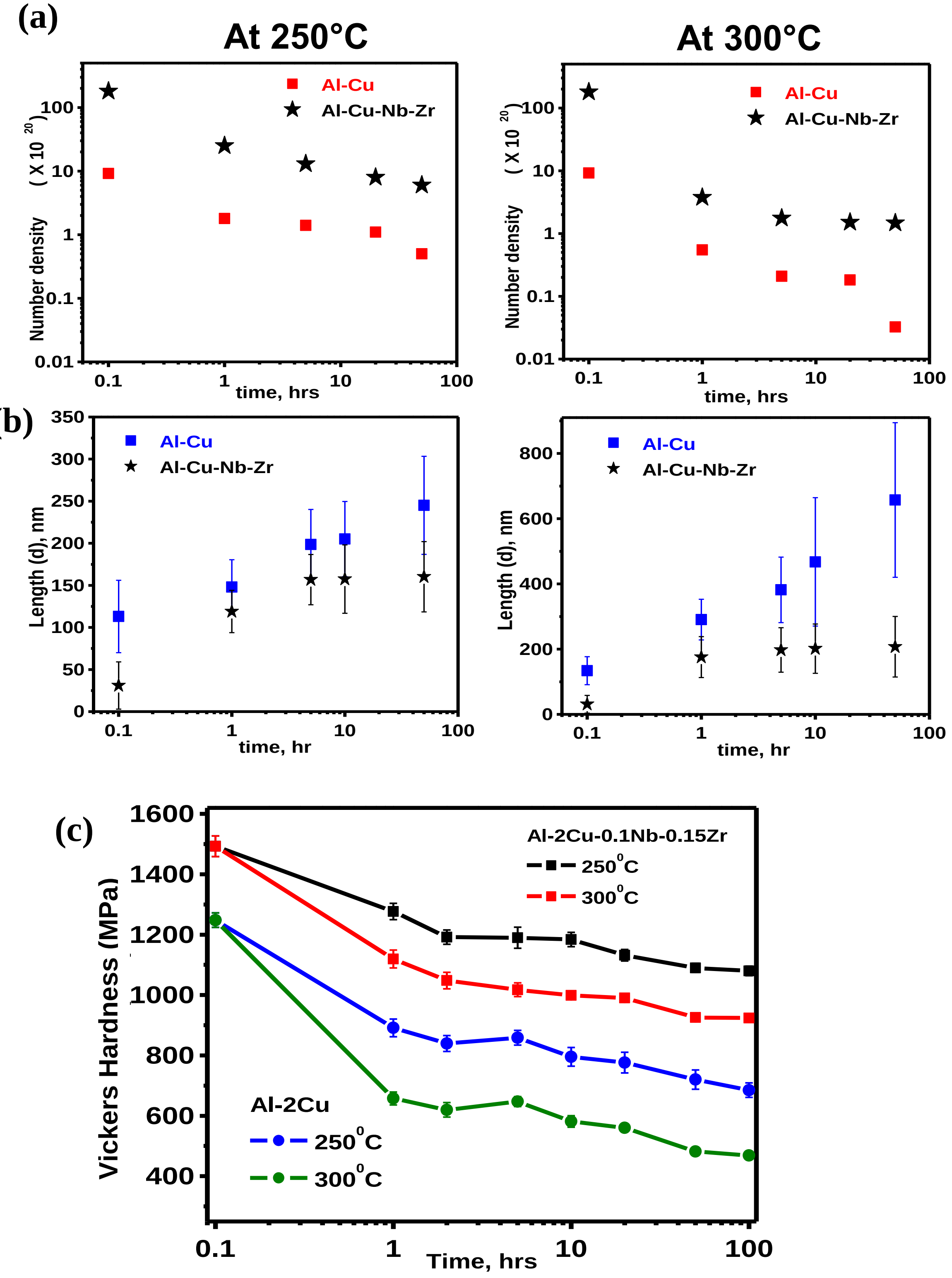

S8: Variation of (a) number density and (b) plate precipitate length with time for both peak aged (at 190°C) Al-2Cu and Al-2Cu-0.1Nb-0.15Zr alloy on exposure to 250°C and 300°C (c) Hardness vs time plot for both peak aged (at 190°C) Al-2Cu and Al-2Cu-0.1Nb-0.15Zr alloy on exposure to 250°C and 300°C

S9: Supplement 9

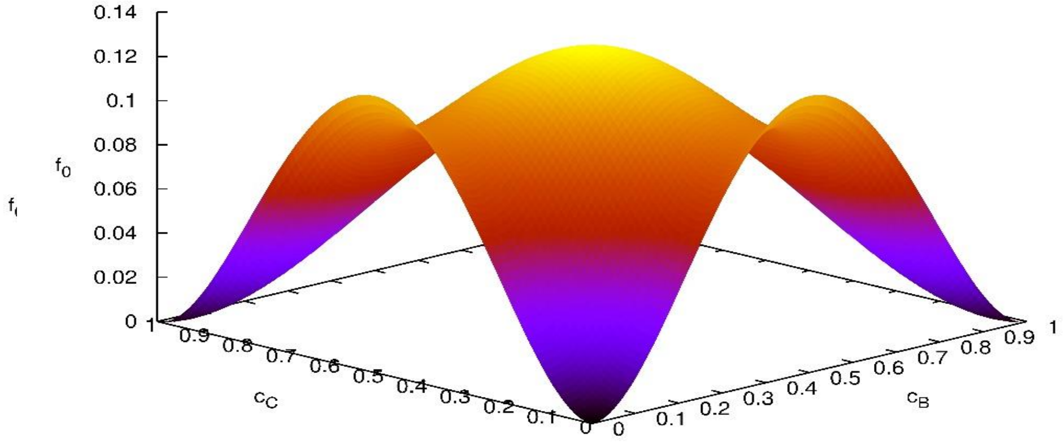

S9: Bulk free energy surface as a function of c_B and c_C showing minima corresponding to the disordered matrix (α), ordered $L1_2$ precipitates (β) and one orientational variant of ordered θ''. Note that θ'' has three orientational variants.
